# Communication Aspects of Visible Light Positioning (VLP) Systems Using a Quadrature Angular Diversity Aperture (QADA) Receiver

**DOI:** 10.3390/s20071977

**Published:** 2020-04-01

**Authors:** Mohammed M. A. Mohammed, Cuiwei He, Stefanie Cincotta, Adrian Neild, Jean Armstrong

**Affiliations:** 1 Department of Electrical and Computer Systems Engineering, Monash University, Victoria 3800, Australia; mohammed.ma.mohammed@monash.edu (M.M.A.M.); cuiwei.he@monash.edu (C.H.); jean.armstrong@monash.edu (J.A.); 2 Department of Mechanical and Aerospace Engineering, Monash University, Victoria 3800, Australia; adrian.neild@monash.edu

**Keywords:** ACO-OFDM, aperture, optical communication, photoreceiver, visible light positioning (VLP)

## Abstract

Visible light positioning (VLP) is a promising indoor localization system in which light emitting diode (LED) luminaires are used as positioning beacons. Data communication is an essential aspect of any VLP system, as each luminaire must transmit information about its own location to the receiver. The quadrature angular diversity aperture (QADA) is a new receiver designed specifically for VLP systems using angle-of-arrival estimation. Previous QADA research has focused only on positioning and assumed error-free communication. In this paper, we investigate, via simulations and experiment, the actual communication characteristics of a VLP system that uses a QADA receiver. We calculate the signal-to-noise ratio and bit-error-rates for a range of scenarios and demonstrate the impact of the dimensions of the receiver. We show that reliable communication is assured in typical operating scenarios, proving that communication will not be a limiting factor when using QADA in VLP systems.

## 1. Introduction

Interest in visible light positioning (VLP) systems has witnessed a dramatic increase in recent years [1]. This has been driven by the need for a reliable localization system that works indoors and by the widespread adoption of light emitting diodes (LEDs) for lighting applications [2].

There has been a long history of indoor positioning techniques using radio frequency (RF) technologies, with many excellent survey papers on the topic [3]. The most commonly used RF technologies are WiFi, Bluetooth low energy (BLE) and ultra-wideband (UWB). In WiFi positioning, the existing access points that are commonly deployed in buildings for wireless communication, are also used as positioning beacons [4,5]. This contrasts with BLE positioning where a dedicated infrastructure is deployed, typically using battery operated beacons [6,7]. Bluetooth systems are commonly used for location-based services and point of interest broadcast. UWB systems also need to deploy specialized transmitters, but unlike BLE, the receivers are not commonly available in current consumer electronic devices like smartphones [8,9,10].

Compared to other indoor positioning technologies, VLP has several important characteristics. Indoor positioning technologies that use radio waves, such as WiFi and Bluetooth, tend to be heavily impacted by multipath propagation. This means that these technologies often use fingerprinting techniques where measurements are gathered during an offline stage to generate a map [4,6,11]. In operation, position estimates are based on matching the received signals to the previously generated map. Due to the ever-changing environment inside, it is challenging to keep the maps up to date and accuracy can be degraded. Light-based systems do not suffer as much from issues related to reflections [12], thus, it is possible to use line of sight techniques, such as received signal strength (RSS), time of arrival (TOA) and angle of arrival (AOA), in VLP. This means very accurate and reliable positioning is possible. Although UWB does not suffer as much from the multipath problem, it does require expensive dedicated infrastructure. VLP only needs minor modifications to existing infrastructure as almost all indoor areas are already illuminated.

Communication is a key part of VLP systems where each luminaire serves as a beacon for indoor positioning and must transmit information about its location, such as its local coordinates. This can be achieved because LEDs can be switched at very high speeds, and this facilitates their dual usage for illumination and data transmission [13]. In VLP systems, a receiver must combine two capabilities. It must detect the source of light so that the location of the receiver relative to the luminaire can be found. In addition, it must also decode a signal from the luminaire which gives information on the location of the luminaire. Combined, this information yields the location of the receiver.

The angular diversity aperture (ADA) receiver was initially developed for use in visible light communication (VLC) systems [14]. An ADA is a directional optical receiver that consists of multiple receiving elements, each of which is composed of a photodiode (PD) placed below an aperture. It has been shown that when used for VLC systems, the ADA is capable of providing a wide field of view (FOV) and excellent angular diversity within a compact and flat structure, which makes it easily embedded into modern portable devices, such as smartphones. The ADA has a flexible structure that can be tailored to specific applications by varying its parameters [15]. For example, the ADA design can be optimized for use in cellular or multiple-input multiple-output (MIMO) systems.

A related receiver, the quadrature angular diversity aperture (QADA) receiver, has been specifically designed for VLP systems [16,17]. For QADA to be used in VLP systems, it must perform two tasks, it must calculate the angle of arrival, and decode the signal which is transmitted. The positioning accuracy of a VLP system using a QADA has been designed for the former, but the latter has not been examined yet. This paper presents the first analysis and experimental demonstration of the communications performance of a VLP system using a QADA receiver.

The importance of angular diversity receivers in VLP systems is due to the key advantages of AOA-based positioning systems compared with systems using other techniques such as RSS and TOA [1,18]. VLP systems using RSS depend on the receiver having accurate knowledge of the transmission pattern and the transmit power of each luminaire, while systems using TOA depend on very accurate synchronization; in the order of nanoseconds. Systems using AOA have none of these limitations. They do however require a receiver that can determine the AOA of light from each beacon luminaire.

Several different forms of AOA receivers have been described in the literature [19,20,21]. In some, angular diversity is achieved using a 3-D structure [20], where multiple PDs face in different directions. However, a 3-D structure of this form is not likely to be convenient, especially if the receiver is to be integrated into handheld devices. In others, angular diversity is achieved by using a camera [19]. Despite many innovative approaches, such as the use of the rolling shutter feature of many digital cameras, the rate of data that can be received using a camera in VLP is strictly limited. ADA-based receivers have the potential to overcome these disadvantages. They provide angular diversity within a compact structure and can receive high-speed data.

It was shown in [22] that, theoretically, the original form of ADA receiver [14] can also be used for VLP systems. However, certain unrealistic assumptions were made in [22]; for example that the PDs were identical to each other and that the dimensions of the aperture and the PD were precise and perfectly aligned with each other. In practice, such constraints are hard to meet. The QADA relaxes some of these constraints. The optical front-end of a QADA receiver consists of a single quadrant PD, with four closely matched quadrants (fabricated in one process), placed below a single aperture [16]. To further improve the positioning accuracy of the QADA, the QADA-plus was introduced [23]. The QADA-plus is a two-stage receiving system that combines the QADA with a camera, but it still uses the QADA stage as the data receiver [24].

Communication is an essential aspect of VLP and this must be considered when designing any VLP system [25,26]. The earlier work on VLP systems that uses QADA receiver considered only the positioning aspects [16,17,23,24,27]. In this paper, we investigate its communication characteristics (Please note that this paper is **not** about investigating the positioning performance as this has been extensively studied in previous work. In this paper, we **only** focus on the characterization of the communication properties of a VLP system using a QADA receiver).

Several different types of modulation can be used in VLP systems [28]. In this paper, asymmetrically clipped optical orthogonal frequency division multiplexing (ACO-OFDM) is used [29]. ACO-OFDM is a simple and power-efficient modulation scheme. Using ACO-OFDM allows us to clearly demonstrate the performance of the QADA receiver without having to consider other parameters, such as DC-bias or clipping distortion which are required to fully specify other modulation formats [30].

We also investigate the performance gains that can be achieved by exploiting the diversity present in the signals received by each of the quadrants of the QADA. While diversity has been extensively applied in RF systems, there is relatively little work on diversity in VLC systems. This is probably because VLC systems provide little *spatial* diversity at the receiver unless the receiving elements are spaced far apart [31]. In contrast *angular* diversity has been shown to provide significant gain [32,33].

Several major contributions have been presented in this paper and are summarized as follows:

1. The communication aspects of VLP systems that use QADA are characterized for the first time. To do this we first calculate the channel gain as a function of the receiver properties. We then analyze the signal-to-noise ratio (SNR) performance of each quadrant individually. The performance is investigated at different locations and for different orientations of the receiver. The results show that reliable communication can be achieved in VLP systems that use the QADA receiver.
2. The diversity among the four quadrants of the receiver is described for the first time, and it is shown how this diversity can be exploited to improve performance. This is done by considering three different techniques. In the first, the signal from the quadrant with the maximum channel gain is selected. In the second and the third, equal gain combining (EGC) and maximum ratio combining (MRC) are applied to the signals of the four quadrants.
3. The relation between the aperture height in a QADA and its FOV is analyzed. In this new analysis, two QADAs with two aperture heights are considered. The communication FOV and positioning FOV are clearly described and are shown to be different.
4. The communication performance of a VLP system using a QADA is investigated in a typical room equipped with four luminaires.
5. Experimental results confirm the very reliable communication performance of the QADA and demonstrate the importance of diversity combining.

The result is a comprehensive analysis of the communications properties of a VLP system using a receiver specifically designed for positioning.

## 2. QADA Optical Front-End

**Figure 1:**
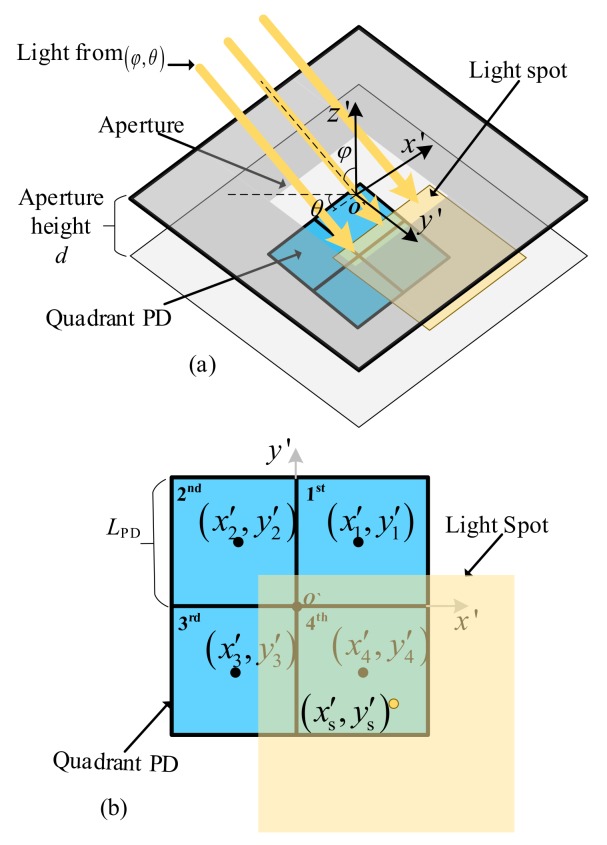
QADA (**a**) receiver structure; (**b**) an example of the overlap between the light spot and the quadrant PD.

The optical front-end of a QADA receiver is shown in Figure 1a. It consists of a quadrant PD, shown in blue located directly below an aperture. The aperture is made in an opaque screen that is parallel to the plane on which the PD is placed. Light passing through the aperture falls on the quadrant PD creating a light spot, shown in yellow. In Figure 1a the light is coming from a particular direction denoted by $\left(\varphi ,\theta \right)$, where φ is the incident angle and θ is the polar angle. Based on the direction of the light source, the light spot may overlap with the quadrant PD beneath it. Figure 1b shows an example of the overlap between the light spot and the PD. The aperture has the same size and shape as the PD and the shape and size of the light spot match that of the aperture. A square quadrant PD and a square aperture are chosen. This is similar to the design in [17] in which the positioning performance of a VLP system using QADA was studied. Each quadrant in the PD has its own output, thus four signals are generated at the output of the PD.

The locations of the light spots and the locations of the quadrants are defined in the coordinate system, $x^{\prime }y^{\prime }z^{\prime }$, with the origin, $O^{\prime }$, at the center of the aperture as shown in Figure 1b. In these coordinates, the center of the *k*-th quadrant is given by

(1) $$\begin{array}{cc} \left({x^{\prime }}_{k},{y^{\prime }}_{k},{z^{\prime }}_{k}\right)= & (\frac{L_{\mathrm{PD}}}{\sqrt{2}}\cos\left(k\frac{\pi }{2}-\frac{\pi }{4}\right),\\ \; & \frac{L_{\mathrm{PD}}}{\sqrt{2}}\sin\left(k\frac{\pi }{2}-\frac{\pi }{4}\right),-d) \end{array}$$

where $L_{\mathrm{PD}}$ is the length of the quadrant of the PD and *d* is the aperture height. The center of the light spot is given by

(2) $$\left({x^{\prime }}_{s},{y^{\prime }}_{s},{z^{\prime }}_{s}\right)=\left(d\cos\theta \tan\varphi ,d\sin\theta \tan\varphi ,-d\right)$$

## 3. Communication Characteristics of a QADA Receiver

### 3.1. Channel Gain

The channel gain of the optical channel in a VLC or VLP system is the ratio between transmitted and received optical powers. The transmitter used in this paper is assumed to be a Lambertian source with order *m* [34]. In this paper, and in line with other work [33], only the line of sight component is considered. This is because of the properties of VLC. In VLC systems, unlike RF systems, the multipath components are very small and are negligible compared with the line of sight component [34]. Additionally, our previous work [14] has shown that due to their directionality, the communication performance of aperture receivers is mostly unaffected by the diffuse component. Therefore, the channel gain for the *k*-th quadrant of the quadrant PD is given by

(3) $$h_{k}=\frac{m+1}{2\pi l^{2}}A_{o}^{\left(k\right)}\cos^{m}\phi \cos\varphi \;\;$$

where *l* is the distance between the luminaire and the receiver, $A_{o}^{\left(k\right)}$ is the overlap area between the light spot and the *k*-th quadrant of the PD, and ϕ is the emission angle.

The overlap area between the light spot and each quadrant is given by

(4) $$\begin{array}{cc} A_{o}^{\left(k\right)}= & \max\{0,\min\left\{{x^{\prime }}_{s}+L_{\mathrm{PD}},{x^{\prime }}_{k}+\frac{L_{\mathrm{PD}}}{2}\right\}-\\ \; & \max\left\{{x^{\prime }}_{s}-L_{\mathrm{PD}},{x^{\prime }}_{k}-\frac{L_{\mathrm{PD}}}{2}\right\}\}\times \\ \; & \max\{0,\min\left\{{y^{\prime }}_{s}+L_{\mathrm{PD}},{y^{\prime }}_{k}+\frac{L_{\mathrm{PD}}}{2}\right\}-\\ \; & \max\left\{{y^{\prime }}_{s}-L_{\mathrm{PD}},{y^{\prime }}_{k}-\frac{L_{\mathrm{PD}}}{2}\right\}\} \end{array}$$

**Figure 2:**
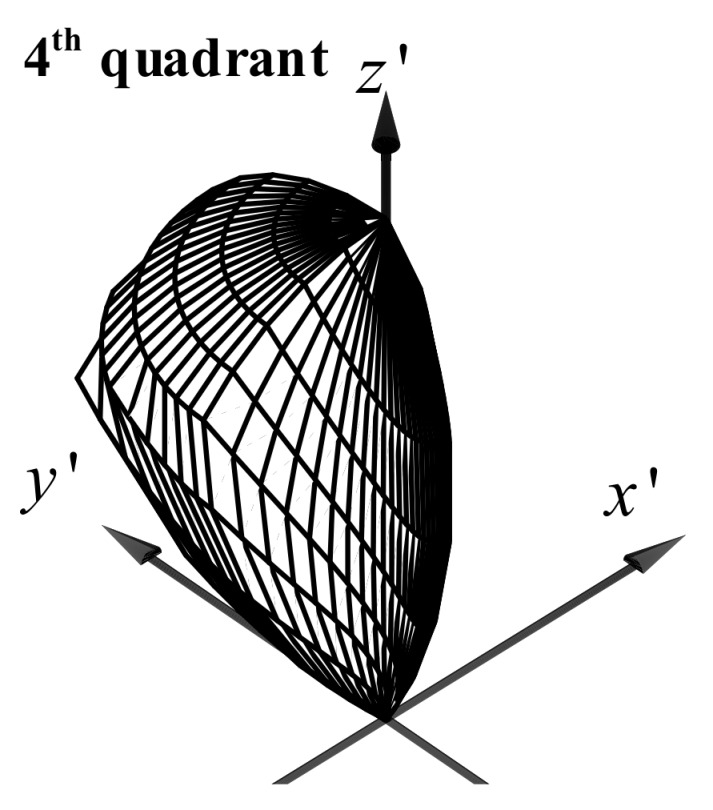
Normalized 3-D channel gain of the $4^{\mathrm{th}}$ quadrant of the QADA receiver. The gain of the other three quadrants will look similar, but ‘point’ in different directions

Figure 2 illustrates the normalized 3-D channel gain plot for the fourth quadrant of the PD in QADA when $d=L_{\mathrm{PD}}$. In this plot, the normalized channel gain for the light coming from a specific direction is represented by the radial distance from the corresponding point on the plot to the origin. It shows that each quadrant of the PD in a QADA has its own specific range of directions from which the light can be received with high gain.

### 3.2. Field of View

**Figure 3:**
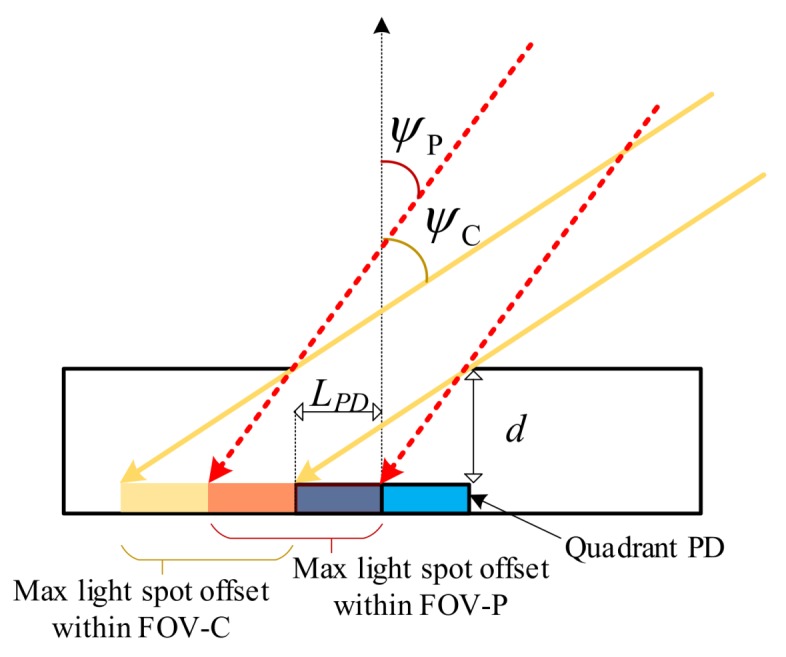
Difference between the FOV-C and the FOV-P of the QADA receiver.

To understand both the communications and positioning characteristics of a VLP system using a QADA, it is important to define two different fields of view. This is because, for positioning, light from a given transmitter must reach all four quadrants of the PD, whereas for communications it is sufficient for light to reach a single quadrant. To make clear the difference, we introduce new terminology. FOV-C is used to designate the FOV for communications and FOV-P is used to designate the FOV for positioning. Figure 3 shows the difference between FOV-C and FOV-P. It shows that the FOV-P is smaller than FOV-C. The FOV-C is defined by

(5) ψC=tan-12LPD2LPDdd

and the FOV-P is given by

(6) ψP=tan-1LPD2LPDdd

**Figure 4:**
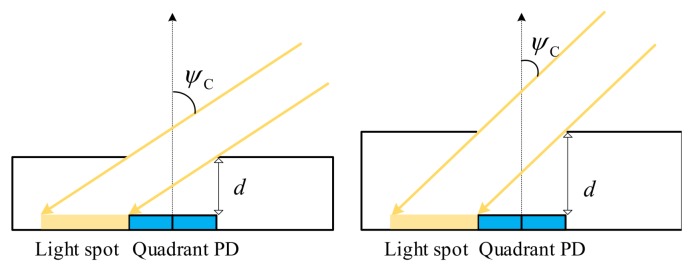
Relation between the FOV-C of the QADA receiver and the aperture height.

The FOV of a QADA depends mainly on ratio $L_{\mathrm{PD}}/d$. Figure 4 shows how increasing the aperture height reduces FOV-C which makes the receiver more directional. This also applies to FOV-P.

### 3.3. Noise

Two sources of noise are considered: shot noise and thermal noise. Therefore, the total noise is the sum of both. The shot noise is modelled as Gaussian noise. The spectral density of the shot noise of each quadrant is given by

(7) $$N_{0}=2qRp_{n}A_{k}\Delta \lambda$$

where *q* is the electron charge, *R* is the PD responsivity, $p_{n}$ is the spectral irradiance, $A_{k}$ is the area of each quadrant in the PD, and Δλ is the optical bandwidth. The area of one quadrant in a square PD is given by $A_{k}={L_{\mathrm{PD}}}^{2}$. The spectral irradiance depends on the level of illuminance and the spectral characteristics of the light source. Typically, work on VLP or VLC uses a spectral irradiance of $p_{n}=6\;\mu W/\left(cm^{2}\cdot \mathrm{nm}\right)$, which represents a worst-case scenario when the receiver is close to a window in a daylight environment [35]. Instead, in this paper, in order to provide more realistic results, the spectral density of the background light is $p_{n}=0.29\;\mu W/\left(cm^{2}\cdot \mathrm{nm}\right)$. This represents a case of the spectral irradiance caused by LED luminaires that produces typical level of illumination. More details on how this value is derived can be found in Appendix B.

The thermal noise spectral density is calculated based on the noise equivalent power (NEP), which is the incident light level required to generate a current equal to the thermal noise current [36]. NEP is used by the manufacturers of PDs to express the level of noise and can be found in most datasheets [37]. The spectral density of the thermal noise is given by

(8) $$N_{\mathrm{Thermal}}={\left(\mathrm{NEP}\left(\lambda_{0}\right)\times R\left(\lambda_{0}\right)\right)}^{2}$$

where $\lambda_{0}$ is the wavelength at which the NEP is measured. The value of NEP used in this paper is $\mathrm{NEP}=1.4\times 10^{-14}\;W/\sqrt{\mathrm{Hz}}$, which is taken from the datasheet of the quadrant PD (Hamamatsu S5980 [38]). This is the PD used in the experiment in Section 6.

## 4. SNR Analysis of a QADA Receiver

To analyze the SNR performance of a QADA receiver, two different receiver designs with different FOVs and two different transmitter radiation patterns are used. We also show how the performance can be improved by combining the outputs from different quadrants.

**Figure 5:**
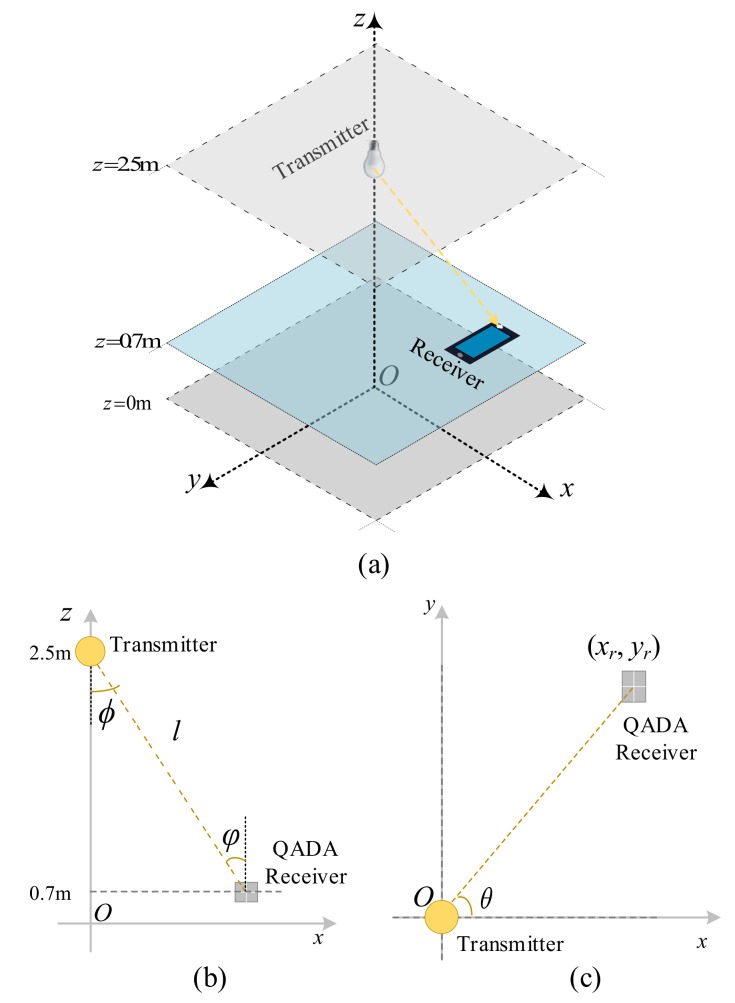
Communication system with a single transmitter at the center (**a**) a 3-D view of the configuration with a single transmitter and a QADA receiver; (**b**) a side view of the configuration; (**c**) a top view of the configuration.

Figure 5 shows the receiver and the transmitter coordinate system xyz. This is different from the coordinate system used within the QADA front-end so that the effect of different receiver orientations can be studied. In this section, a single transmitter with fixed optical power is considered. It is placed directly above the origin, *O*. To make the results in this section consistent with the next section, the transmitter and the receiver are placed at z=2.5m and z=0.7m, which represent typical ceiling and table heights in an indoor scenario.

**Figure 6:**
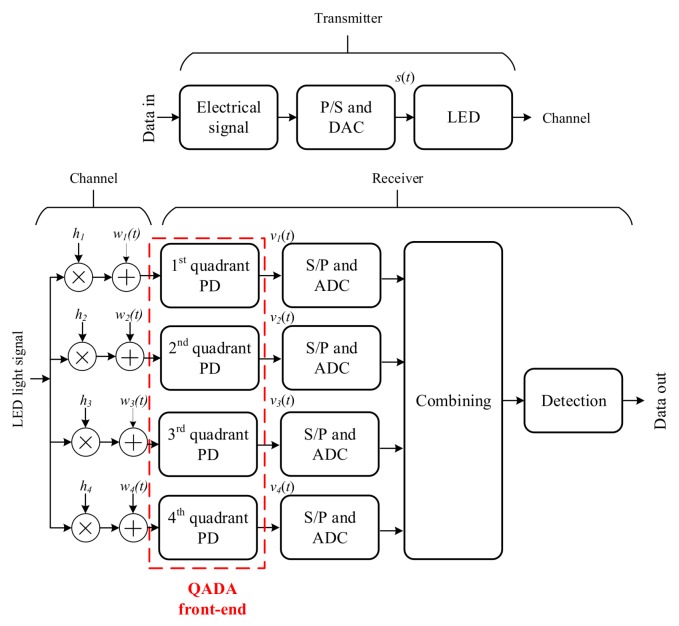
System block diagram of the transmitter and the QADA receiver. P/S is parallel to serial and S/P is serial to parallel.

The block diagram of the main components in the transmitter and the receiver are shown in Figure 6. An electrical signal that carries the data is generated at the transmitter, which is then used to modulate the intensity of the LED. At the receiver, the optical signal is converted back to an electrical signal by the quadrant PD. At the output of the optical front-end, four signals are generated, each corresponding to one of the quadrants. After the analog-to-digital converter (ADC), the received signal from each quadrant *k* is given by

(9) $$v_{k}\left(t\right)=h_{k}s\left(t\right)+w_{k}\left(t\right)$$

where $s\left(t\right)$ is the transmitted signal, $w_{k}\left(t\right)$ is the noise, and $h_{k}$ is the channel gain. The electrical SNR of the signal received from the *k*-th quadrant is expressed as

(10) $${\mathrm{SNR}}_{k}=\frac{h_{k}^{2}E\left[{\left|s\left(t\right)\right|}^{2}\right]}{E\left[{\left|w_{k}\left(t\right)\right|}^{2}\right]}$$

where $E\left\{\cdot \right\}$ is the expected value. The transmitted data can be recovered directly from one of the quadrants; however, this does not take advantage of the potential benefit of the four quadrants.

The total received signal from all quadrants is expressed as

(11) $$v\left(t\right)=hs\left(t\right)+w\left(t\right)$$

where $v={\left[v_{1}\left(t\right),v_{2}\left(t\right),v_{3}\left(t\right),v_{4}\left(t\right)\right]}^{T}$, $w={\left[w_{1}\left(t\right),w_{2}\left(t\right),w_{3}\left(t\right),w_{4}\left(t\right)\right]}^{T}$, and $h={\left[h_{1},h_{2},h_{3},h_{4}\right]}^{T}$. Combining can be used to benefit from the diversity in the four signals. In general, the SNR after combining is given by

(12) $$\mathrm{SNR}=\frac{{\left|\alpha^{T}h\right|}^{2}E\left[{\left|s\left(t\right)\right|}^{2}\right]}{E\left[{∥w\left(t\right)∥}^{2}\right]}$$

where $\alpha ={\left[\alpha_{1},\alpha_{2},\alpha_{3},\alpha_{4}\right]}^{T}$ and $\alpha_{k}$ is the weighting factor for the signal from the *k*-th quadrant.

There are multiple combining methods that can be applied and thus improve the performance. The first is the selection combining where the quadrant with the highest power is chosen. The weighting factor, in this case, is given by

(13) $$\alpha_{k}=\left\{\begin{array}{c} 1,\;\;\;\;\;\;\mathrm{if}\;E\left[{\left|v_{k}\left(t\right)\right|}^{2}\right]=\max_{n\in [1,\cdots ,4]}\left\{E\left[{\left|v_{n}\left(t\right)\right|}^{2}\right]\right\}\\ 0,\;\;\;\;\mathrm{otherwise} \end{array}\right.$$

The SNR can be improved if the four signals are added together. In its simplest form, the signals from all quadrants are combined using EGC. The weighting factor using EGC is $\alpha_{k}=0.5$ for all *k*. EGC is only optimum when the signals have the same channel gain, which is not usually the case for a QADA receiver. To fully use the diversity of the signals, the combining should be proportional to the channel gains to maximize the SNR. To do that, we use the well-known MRC algorithm [39]. The weighting factor using MRC is given by

(14) αk=hkhk∑k=14hk2∑k=14hk2

The SNR of the signal after MRC combining is given by

(15) $$\mathrm{SNR}=\frac{\sum_{k=1}^{4}{\left|h_{k}\right|}^{2}E\left[{\left|s\left(t\right)\right|}^{2}\right]}{E\left[{∥w\left(t\right)∥}^{2}\right]}$$

### Simulation Results and Discussion

The parameters used in the simulations are summarized in Table 1. The transmitted optical power of the luminaire is set at 1 W and luminaires with two different Lambertian orders, m=1 and m=4, are considered. This is to represent both a non-directional and a directional source. The QADA receiver has a square aperture that has the same size as the PD. Two values for the aperture height are used: d=2.5mm and d=5mm. These correspond to FOV-Cs of $63.4^{\circ }$ and $45^{\circ }$, respectively. The parameters related to the quadrant PD are chosen to reflect the commercially available Hamamatsu S590 that is used in the experimental work, detailed in Section 6.

**Figure 7:**
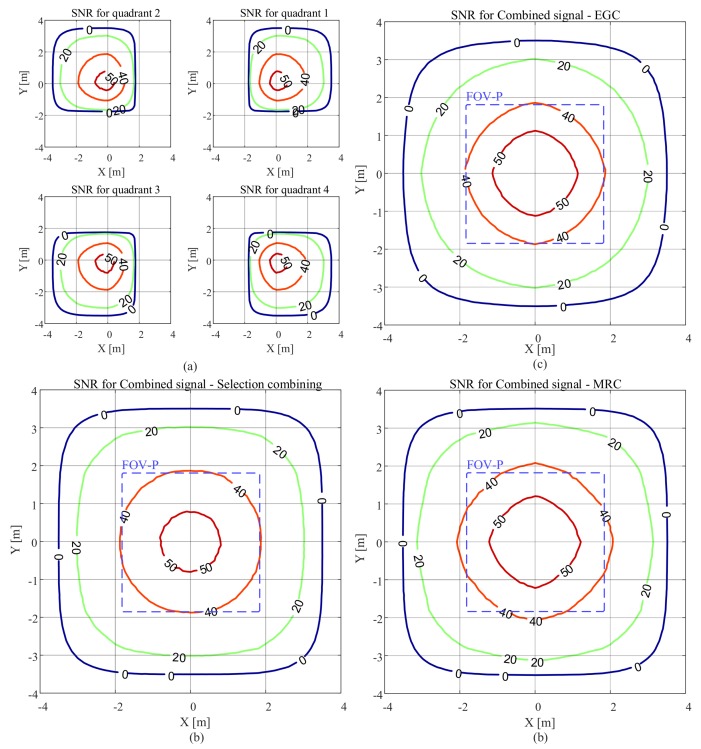
SNR in dB versus QADA location in an area equipped with a single transmitter with m=1 and with the receiver parallel to the plane on which the luminaire is located with a FOV-C of $63.4^{\circ }$ for (**a**) each quadrant, (**b**) the combined signal using selection combining, (**c**) the combined signal using EGC, (**d**) the combined signal using MRC.

The SNR results as a function of the location of the receiver as *x* and *y* are varied are presented in Figure 7. Here, m=1, FOV-C $=63.4^{\circ }$, and the receiver is parallel to the plane on which the luminaire is located. The SNR contour plots for each quadrant of the QADA receiver are shown in Figure 7a. For each quadrant, the SNR is relatively high in some locations. For example, for the fourth quadrant, the SNR is more than 20 dB, for values of *x* between −1.8 m and 3 m and values of *y* between −3 m and 1.8 m. This is because for this region the channel gain is large as shown in Figure 2. Figure 7b shows the SNR contour plots when the quadrant with the maximum power is selected. Now, as long as the luminaire is within the FOV-C of the receiver, the SNR is high in most locations.

The SNR can be further improved if the signals from all quadrants are combined as shown in Figure 7c,d. After combining the signals from all quadrants, the SNR improves significantly. Figure 7c shows the results when the EGC is used and Figure 7d shows the SNR results when the MRC algorithm is applied. Using MRC (MRC algorithm is the combining method used for the rest of the paper) for combining gives the best SNR results because it effectively uses the diversity in the signals.

The purple dashed lines represent the region in which light from the luminaire reaches all four quadrants of the QADA so that it can be used for VLP. It is smaller than the overall communication area; however, in this region, the SNR is always high, mostly above 40 dB. We will show in Section 5 that the communications properties will not be a limitation in VLP systems that use QADA.

**Figure 8:**
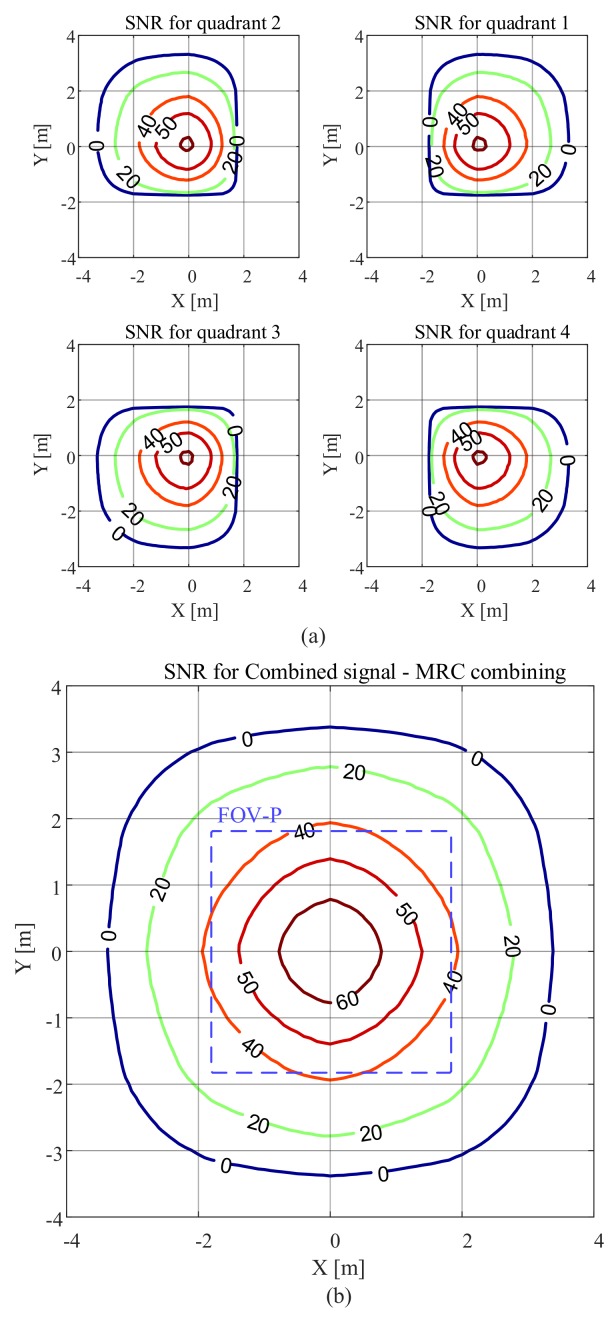
SNR in dB versus QADA location in an area equipped with a single transmitter with m=4 and with the receiver parallel to the plane on which the luminaire is located with a FOV-C of $63.4^{\circ }$ for (**a**) each quadrant; (**b**) the combined signal using MRC.

Figure 8 shows the SNR results when a transmitter with a higher Lambertian order of m=4 is used. In comparison with the results in Figure 7, we can see that the area that has an SNR of less than 0 dB has been reduced and the SNR increased in the area directly below the transmitter. This is because when *m* is increased the radiation pattern becomes more directional.

**Figure 9:**
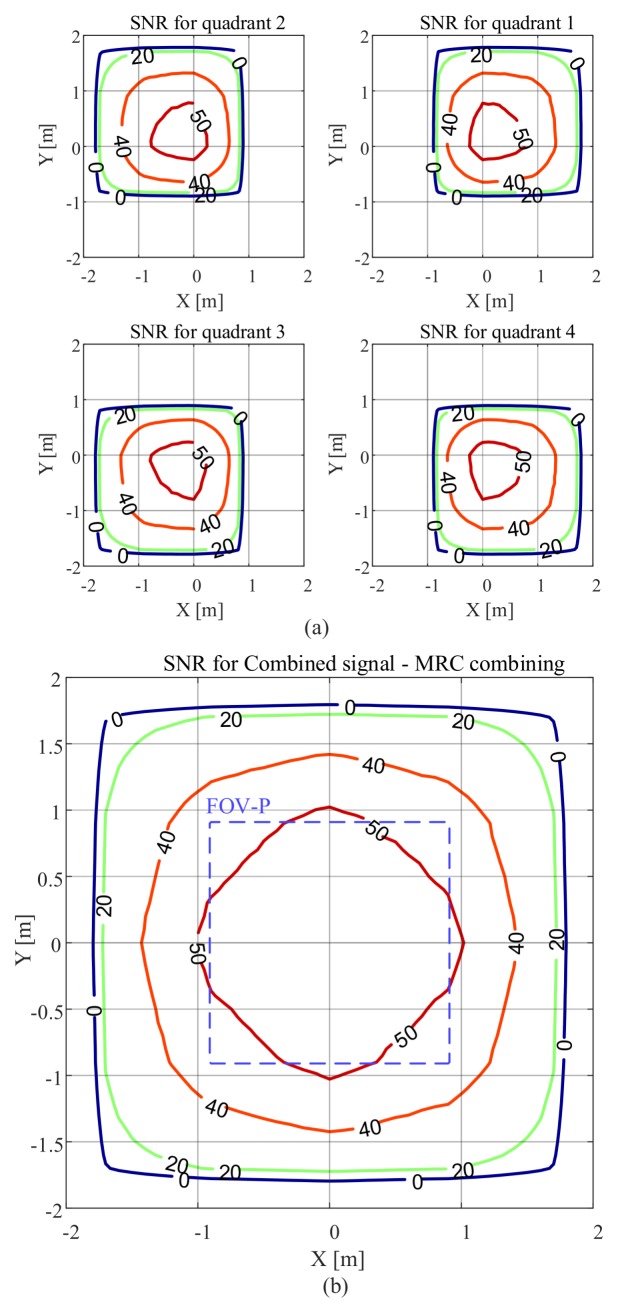
SNR in dB versus QADA location in an area equipped with a single transmitter with m=1 and with the receiver parallel to the plane on which the luminaire is located with a FOV-C of $45^{\circ }$ for (**a**) each quadrant; (**b**) the combined signal using MRC.

Figure 9 illustrates the results for the smaller FOV-C of $45^{\circ }$ and m=1. Please note that the scale is different from Figure 7 and Figure 8 to clearly show the changes of the SNR within the FOV-C. The receiver, within its FOV-C, can still provide very good performance. However, we can see that when the FOV-C is reduced from $63.4^{\circ }$ to $45^{\circ }$, the area where the SNR is less than 0 dB is significantly reduced and the receiver has almost half the coverage area of the receiver with FOV-C of $63.4^{\circ }$. Like Figure 7, the SNR is high within FOV-P.

**Figure 10:**
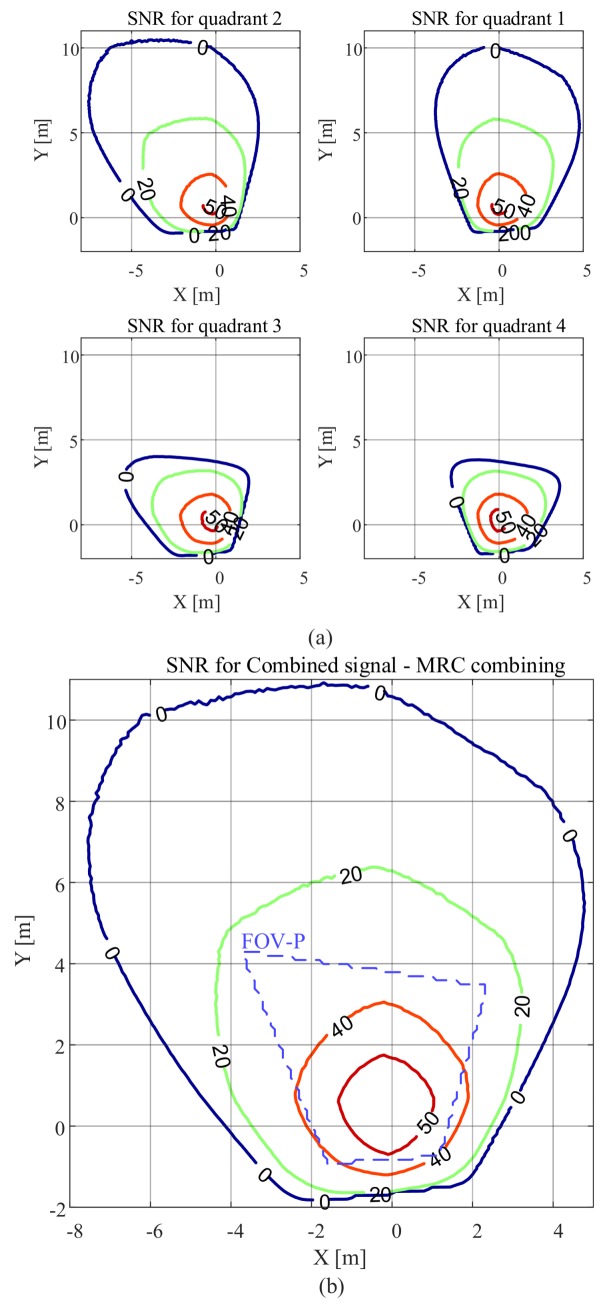
SNR in dB versus QADA location in an area equipped with a single transmitter with m=1 and with the receiver held at an angle (tilted by $20^{\circ }$ along the axis and $5^{\circ }$ along the axis) with a FOV-C of $63.4^{\circ }$ for (**a**) each quadrant; (**b**) the combined signal using MRC.

Finally, to demonstrate the effect of tilt, Figure 10 depicts the SNR performance when the receiver is tilted by 20 degrees along the *x*-axis and 5 degrees along the *y*-axis. This represents a person holding and looking at a smartphone equipped with a QADA. Here we consider a FOV-C of $63.4^{\circ }$ and m=1. The results show that even with a tilt, the QADA still provides good performance across a wide area around the luminaire. Compared with the case when the receiver is parallel to the plane on which the luminaire is located, there is a shift in the SNR contour plots toward the larger values of *y*. When no tilting is applied to the receiver, the SNR is higher than 20 dB, for displacements between −3.2 m and 3.2 m along the *y*-axis (Figure 7). Now at displacements that are less than −1.8 m along the *y*-axis, the SNR is less than 0 dB because, at these locations, most of the light is blocked due to the tilt. Like Figure 7, Figure 8 and Figure 9, the SNR is always high within the FOV-P.

## 5. Communication Performance in a Multi-Luminaire Scenario

**Figure 11:**
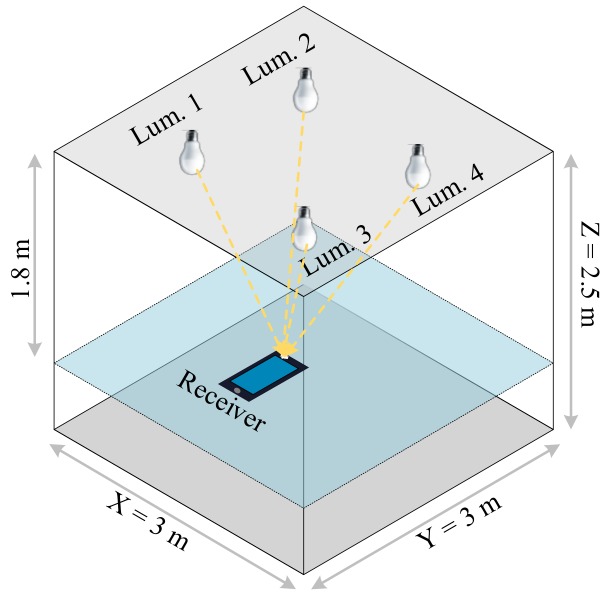
3-D view of the room configuration with four luminaires and a QADA receiver.

In this section, we apply the results obtained from Section IV to a typical indoor scenario, shown in Figure 11. This represents a room that is 3 m in length, 3 m in width, and 2.5 m in height is considered. The origin, *O*, is on the floor at the center of the room. The receiver is placed at a table height of 0.7 m from the floor and is pointing upwards. Four luminaires are placed around the center of the ceiling. They are located at $\left(0.75\;m,\;0.75\;m,\;2.5\;m\right)$, $\left(-0.75\;m,\;0.75\;m,\;2.5\;m\right)$, $\left(-0.75\;m,\;-0.75\;m,\;2.5\;m\right)$, and $\left(0.75\;m,\;-0.75\;m,\;2.5\;m\right)$.

**Figure 12:**
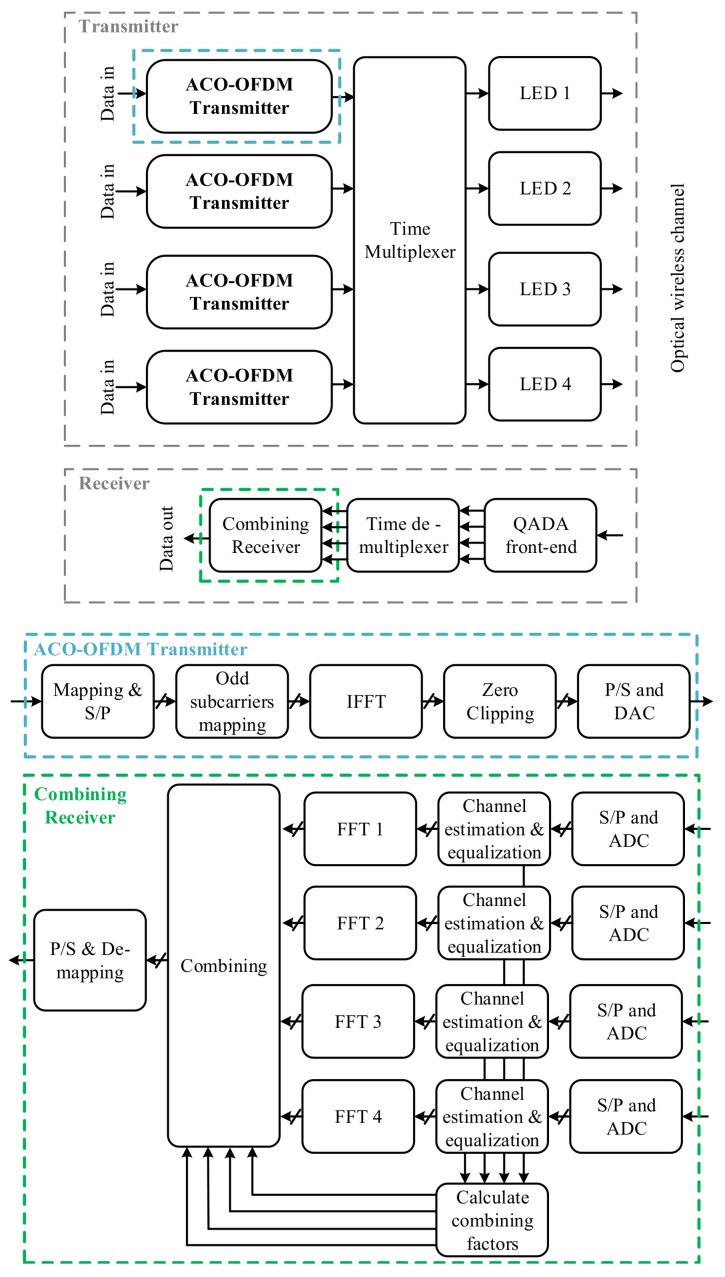
System block diagram of the transmitter and the QADA receiver using ACO-OFDM as the modulation in multi-luminaire scenario and using TDMA as the multiplexing scheme.

The block diagram of the transmitter and the receiver in a multi-luminaire scenario is shown in Figure 12. The data for each luminaire is modulated using an ACO-OFDM modulator. ACO-OFDM with 4-quadrature amplitude modulation (QAM) is used for all luminaires. For each luminaire, the data is first modulated using 4-QAM and then mapped to the odd subcarriers of the OFDM signal. To make the OFDM signal real, Hermitian symmetry is imposed on the input to the inverse fast Fourier transform (IFFT). The signal at the output of the IFFT is then clipped at zero to create a unipolar signal. This discrete signal is converted to a continuous signal using a DAC. Time division multiple access (TDMA) is used to coordinate between luminaires by assigning a specific time slot to each luminaire in which they transmit their signal. This is important to avoid multiple access interference. Finally, the continuous signal is used to directly modulate the intensity of the corresponding LED luminaire.

At the receiver, the quadrant PD converts the optical signal into an electrical signal. The signal from each luminaire is recovered during the corresponding time slot. After estimating the channel gains for a given luminaire, the four signals from that luminaire received during its time slot are equalized and then combined at the output of the FFTs using MRC. Finally, the transmitted data is recovered from the odd subcarriers and then decoded using a maximum likelihood decoder.

### Simulation Results and Discussion

In this section, we present the simulation results for a QADA receiver in a typical room with four luminaires. Each luminaire is transmitting with an optical power of 1.57 W which is enough to provide the required illumination level in the room. See Appendix A for more details.

**Figure 13:**
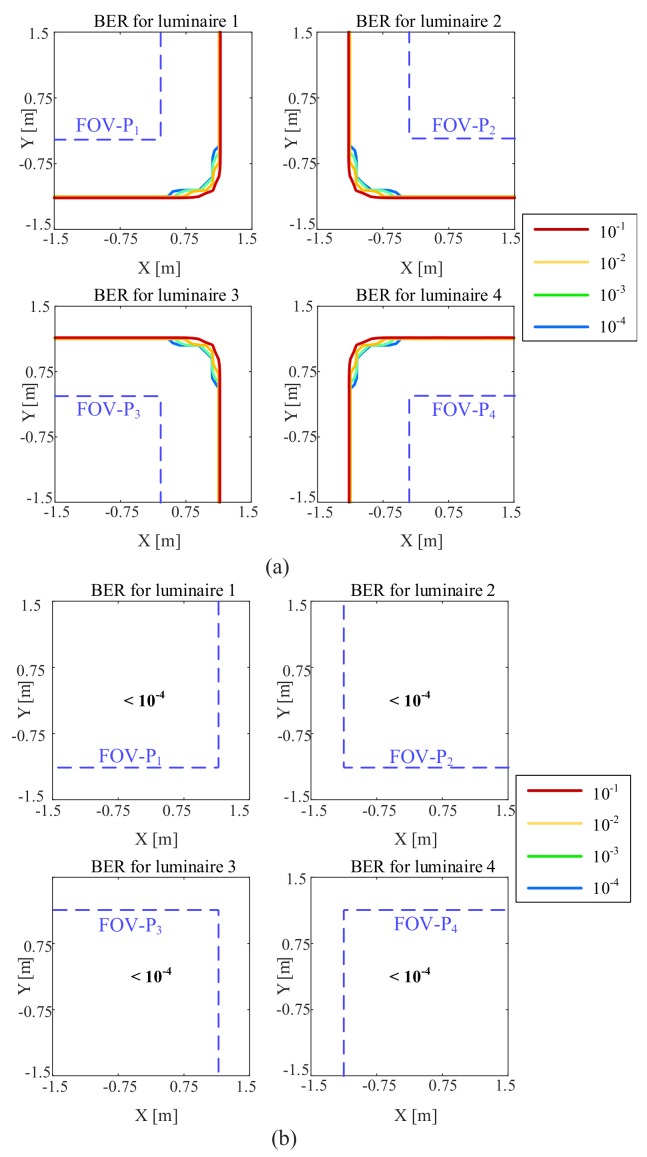
BER versus QADA location in a room equipped with four luminaires when a spectral irradiance of $p_{n}=0.29\;\mu W/\left(cm^{2}\cdot \mathrm{nm}\right)$ and with the receiver parallel to the plane on which the luminaire is located with a FOV-C of (**a**) $45^{\circ }$; (**b**) $63.4^{\circ }$.

Figure 13 shows the BER versus the location of the QADA in the room. As in Section 3, two QADAs with different FOV-Cs are considered. The noise level represents a typical-case scenario of $p_{n}=0.29\;\mu W/\left(cm^{2}\cdot \mathrm{nm}\right)$. We can see that the QADA receiver is capable of providing very good performance in all locations within the room when the FOV-C is $63.4^{\circ }$. The signals from all luminaires are decoded with a BER that is less than $10^{-4}$. Please note that in typical VLP systems, three luminaires are sufficient for the receiver to estimate its location. When the FOV-C is reduced to $45^{\circ }$, as long as the luminaires are within the FOV-C of the receiver, the signals are received with a very low BER. However, because of the smaller FOV-C, in some locations within the room, the receiver is unable to capture the signals from some luminaires. For both values of FOV-C, we can see that the FOV-P for each luminaire is within the low BER area. So, we can conclude that the communication characteristics of a QADA are not going to be a limiting factor when the receiver is used for VLP applications.

## 6. Experiment

### 6.1. Experiment Set-Up

**Figure 14:**
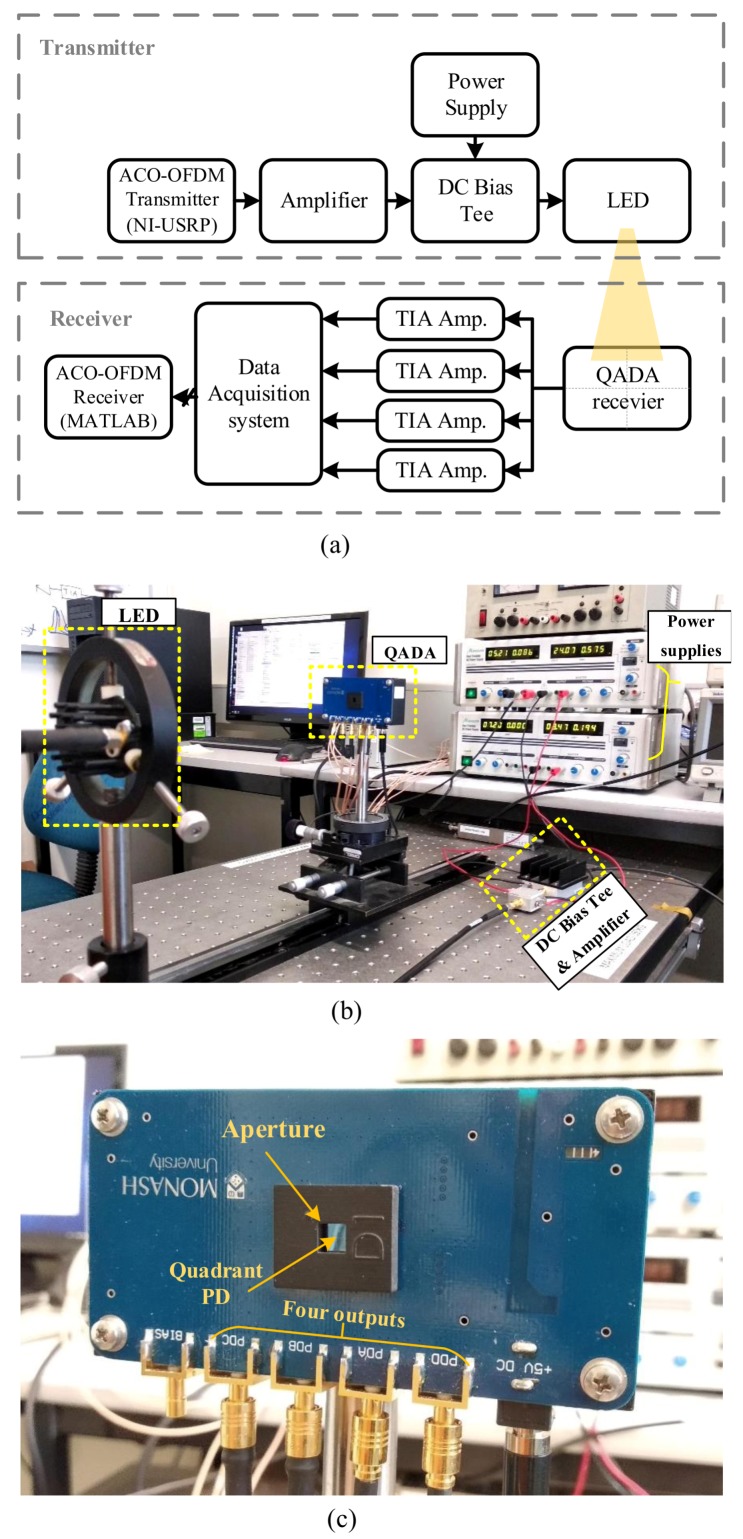
Experimental set-up (**a**) block diagram shows the experimental set-up; (**b**) photo of the experiment; (**c**) photo of the QADA receiver.

In this section, we describe the experiment used to evaluate the communication performance of a VLP system using a QADA. The block diagram of the experimental set-up is shown in Figure 14a and the components used in the experiment are listed in Table 2. The data to be transmitted is modulated using ACO-OFDM, which is generated using a software-defined radio (SDR) module (NI-USRP 2950R). Next, the continuous electrical signal at the output of the SDR system is amplified using an electrical amplifier (Mini-circuits ZHL-32A-S). A DC current source is used to generate a 200 mA current, which is then added to the amplified signal using a DC-bias tee circuit (Mini-circuits ZFBT-4R2GW). The DC current is chosen to be enough to make the LED (Luxeon LXML-PWC2) operate within its linear region. The signal is then used to directly modulate the intensity of the LED. A photo of the experimental set-up is shown in Figure 14b.

**Figure 15:**
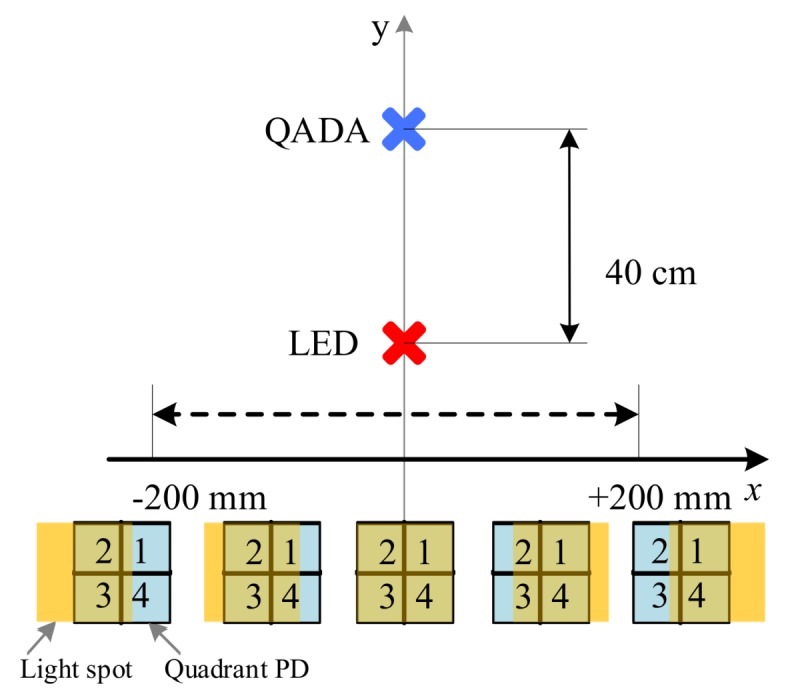
Experiment set-up diagram and the overlap between the light spot and the quadrant PD as the transmitter moves along the *x*-axis.

The QADA converts the optical signal into four electrical signals, each of which represents one of the four quadrants. The QADA is made of a square aperture of size $25\;mm^{2}$ placed in front of a square quadrant PD (Hamamatsu S5980) with a size of $25\;{\mathrm{mm}}^{2}$ at distance d=2.5mm from the aperture and at a distance of 40 cm from the LED. See Figure 15. The QADA front-end is mounted on a custom-built PCB that contains four trans-impedance amplifiers (TIA). The QADA receiver is shown in Figure 14c. The amplified signals are then captured using a data acquisition system (GAGE CSE8389). Finally, the signals are further processed and combined to recover the transmitted data using MATLAB.

The transmitted signal is generated using an ACO-OFDM signal with 16-QAM modulation. 16-QAM modulation is used because when 4-QAM was used the resultant BER was always zero, so it is not suitable to demonstrate the changes of the BER. An FFT length of 256 and a cyclic prefix (CP) length of 32 are considered. The number of subcarriers used is N/4−1; the first subcarrier is not used because it experiences a relatively high attenuation. The total length of the transmitted signal is 2000 OFDM symbols, which represents 512 Kb of data. A baseband daughter board (NI LFTX) is used in the SDR module because the signal required to modulate the LED is a baseband signal. It operates from DC up to 30 MHz. The SDR module replaces the MATLAB signal generation and the arbitrary waveform generator (AWG), which are typically used in similar experiments [40]. Using the SDR for signal generation allows much longer data sequences to be transmitted, and allows most of the processes to be directly implemented and controlled by software. The module used has very high accuracy and thus the hardware induced impairments are small. The USRP 2950R is connected to a personal computer (PC) which runs National Instrument (NI) LabVIEW FPGA 2016. The PC is connected with the module through a PCIeX4 interface card which loads the compiled files from the LabVIEW environment onto the FPGA motherboard. The sampling rate used is 1 MS/s, resulting in an overall data rate of the transmitted signal is approximately 984.4 Kb/s.

### 6.2. Experimental Results and Discussion

**Figure 16:**
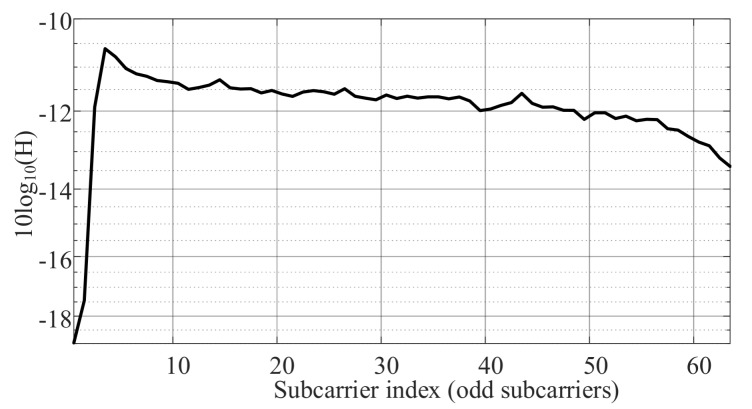
Experiment channel frequency response.

The channel of the system in this experiment is measured at the middle of the *x*-axis. See Figure 16. The values shown represent the frequencies from DC up to 500 kHz. The channel response cuts off at very low frequencies due to the DC-bias-Tee circuit used in this experiment. So, the first data-carrying subcarrier of the ACO-OFDM signal is not used.

**Figure 17:**
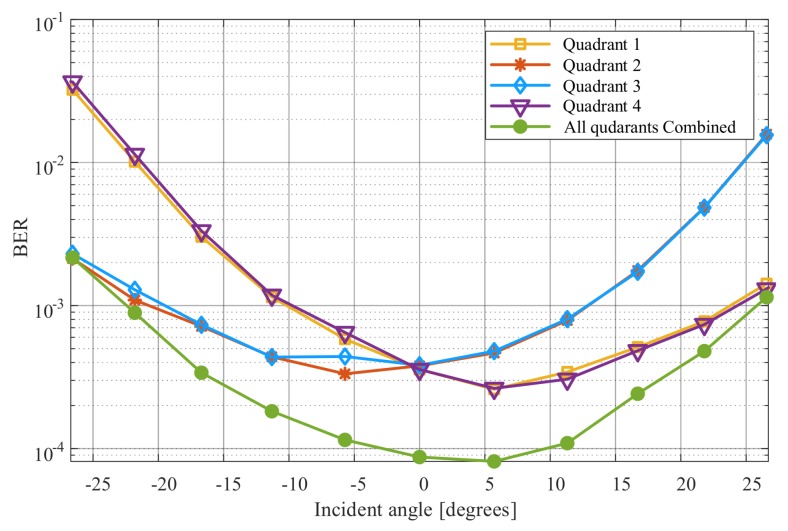
Experimentally measured BERs for each quadrant and for the combined signal using MRC versus the angle of arrival as the transmitter is moved along the *x*-axis.

The BER results for each quadrant and the BER results for the combined signal as a function of the transmitter displacement along the *x*-axis are shown in Figure 17. The transmitter is moved horizontally along the *x*-axis, while the receiver is fixed. This changes two channel parameters simultaneously: the incident angle and the distance between the transmitter and the receiver. The transmitter is moved along the *x*-axis between −200 mm and 200 mm as shown in Figure 15. This corresponds to incident angles between $-26.5^{\circ }$ and $26.5^{\circ }$. The distance is minimum when the transmitter is in the middle and it increases as the transmitter moves to the left or the right.

As the transmitter moves from left to right, the BERs for quadrants one and four decrease until the transmitter reaches the middle and then it starts to slightly increase as it moves to the right. This is because, as the transmitter moves from left to the middle, the light spot partially overlaps both quadrants and when the transmitter is in the middle it fully overlaps both quadrants. After that, the light spot fully overlaps both quadrants as the transmitter moves to the right (see Figure 15). However, as the transmitter moves from the middle to the right, the distance increases which causes the BERs to slightly increase. Similarly, we can describe the BER changes for quadrants two and three; however, for these, the BERs decreases as the transmitter moves from right to left. After combining the four signals using the MRC algorithm, the BER is significantly reduced for most *x*-axis displacements. For example, the BER drops below $10^{-4}$ compared to a BER of $0.5\times 10^{-3}$ without combining at a displacement of 40 mm. This demonstrates the advantage of using diversity combining in improving the performance of the QADA receiver.

## 7. Discussion

There are many trade-offs in the design of the communication aspects of a VLP system using a QADA receiver and most of these are shared by VLC systems in general. However, there are also trade-offs between the communications performance and the positioning performance.

Increasing the area of the PD increases both the received signal power and the shot noise power, and results in an overall improvement of SNR [34]. This increase in SNR is at the cost of a decrease in receiver bandwidth caused by the increased stray capacitance associated with the larger PD. Increasing the area of the PD will also increase the positioning accuracy [16]; however, a bulky receiver is generally not desirable in many applications. Our previous work in [24] provides further detail on other trade-offs related to positioning performance, as well as data on the positioning accuracy of the QADA receiver.

Most modulation schemes that can be used in VLC can also be used in QADA-based VLP. There has recently been substantial interest in different forms of optical OFDM [41]. In the simulations we use 4-QAM ACO-OFDM which is a very power-efficient form of OFDM to show the robustness of the system. The data rate can be increased at the cost of increased BER simply by increasing the ACO-OFDM constellation size and in the experiments 16-QAM ACO-OFDM rather than 4-QAM had to be used before any bit errors were detected. Recently several new modulation schemes based on ACO-OFDM have been developed. These increase the bandwidth efficiency at the cost of power efficiency [42] and all of these could be used in a VLP system but could increase the signal processing complexity of both the transmitter and the receiver.

The data rate required for indoor positioning is, in general, quite low. It depends on precisely what position information is transmitted by the luminaires. For example, whether the global coordinates of the luminaire are transmitted or simply the LED-ID. Another consideration is how often position updates are required: slow moving users require less frequent updates than faster moving users. In scenarios with multiple transmitting luminaires, the available bandwidth must be shared and the data rate available for each luminaire depends on the detailed design of the multiplexing system. In location-based services, where information is broadcast in addition to the positioning information, or where the VLP is combined with VLC, higher data rates may be required.

There are many avenues for future research that can be investigated, including exploring methods capable of achieving higher data rates, evaluating the effect of movement on communications and positioning accuracy and testing the combination of a QADA with other technologies for improved positioning.

## 8. Conclusions

In this paper, we have investigated the communications performance of a VLP system using a QADA receiver. We have shown that the FOV for communications (FOV-C) is different from the FOV for positioning (FOV-P), and that FOV-C is always larger than FOV-P. This is because, to be used in calculating the position of the receiver, the signals from a given luminaire must reach all four quadrants of the receiver quadrant PD, whereas for communications only one quadrant is required.

In the SNR simulations of the VLP system, we assumed that the dominant source of noise is shot noise due ambient light. So that these simulations represent realistic situations, we calculated from first principles typical levels of shot noise when the ambient light is generated by LED lighting. These were shown to be much lower than the worst-case shot noise values often used in the literature. These new values were used in the simulations.

SNR results were presented for the case where a single luminaire is transmitting. This transmitter is pointing directly downwards, and the receiver is placed on a plane 1.8 m below the transmitter and is pointing directly upwards. Results were presented as a function of receiver position for transmitters with two different Lambertian radiation patterns (m=1 and m=4). In general, the SNRs of the signals received by different quadrants of the PD are different. It was shown that this diversity can be exploited to improve the overall SNR of the VLP system. Three different ways of exploiting this diversity were considered: selection combining, EGC and MRC. Expressions were derived for the SNR for EGC and MRC. The simulations showed that all combining methods increase the area in which the signals are received with high SNR and that MRC gives the best performance. For the more directional transmitter (m=4), the area with high received SNR is reduced.

Simulation results of the VLP system were presented for receiver designs with two different FOV-Cs. These showed that reducing FOV-C reduces the area where signals are received with high SNR but increases the SNR for receiver positions directly below the transmitter. Finally, the effect of tilt was explored. The simulations showed that for all the cases considered, the SNR was always above 20 dB within the FOV-P. This demonstrates that the communication properties of the receiver will not be a limitation when a QADA is used in a VLP system.

Most AOA positioning algorithms depend on receiving signals from at least three luminaires. To evaluate the communications performance in a complete positioning system, the BER as a function of position was calculated in a typical 3 m by 3 m room with four luminaires. The transmit power of the luminaires was chosen to meet typical standards for indoor illumination. ACO-OFDM with 4-QAM modulation was used. In all cases a BER less than $10^{-4}$ was achieved within the FOV-P demonstrating again that communications performance is not going to limit the positioning performance of VLP systems that use QADA.

Finally, experimental results were presented. These confirmed that reliable communications can be supported by the QADA and that using diversity combining significantly improves the performance.

## Figures and Tables

**Table 1:** Simulation Parameters.

Parameter	Value
Transmitted optical power, $P_{\mathrm{opt}}$	1 W
Transmitter height from the receiver	1.8m
Background spectral irradiance,	$p_{n}=0.29\;\mu W/\left(cm^{2}\cdot \mathrm{nm}\right)$
Noise equivalent power, NEP	$1.4\times 10^{-14}\;W/\sqrt{\mathrm{Hz}}$
Lambertian order, *m*	1, 4
Optical bandwidth, Δλ	400nm
Electrical bandwidth, *B*	1MHz
Quadrant length, $L_{\mathrm{PD}}$	2.5 mm
Aperture height, *d*	2.5 mm, 5 mm
PD total area, *A*	$25\;{\mathrm{mm}}^{2}$
PD responsivity, *R*	0.4 A/W

**Table 2:** Experimental Components.

Component	Model
Quadrant Photodiode	Hamamatsu S5980
Amplifier	Mini-Circuits ZHL-32A-S
Bias-Tee	Mini-Circuits ZFBT-4R2GW
Software-Defined Radio	National Instruments USRP 2950R
Data acquisition system	Gage CSE8389
LED	Luxeon LXML-PWC2

**Figure A1:**
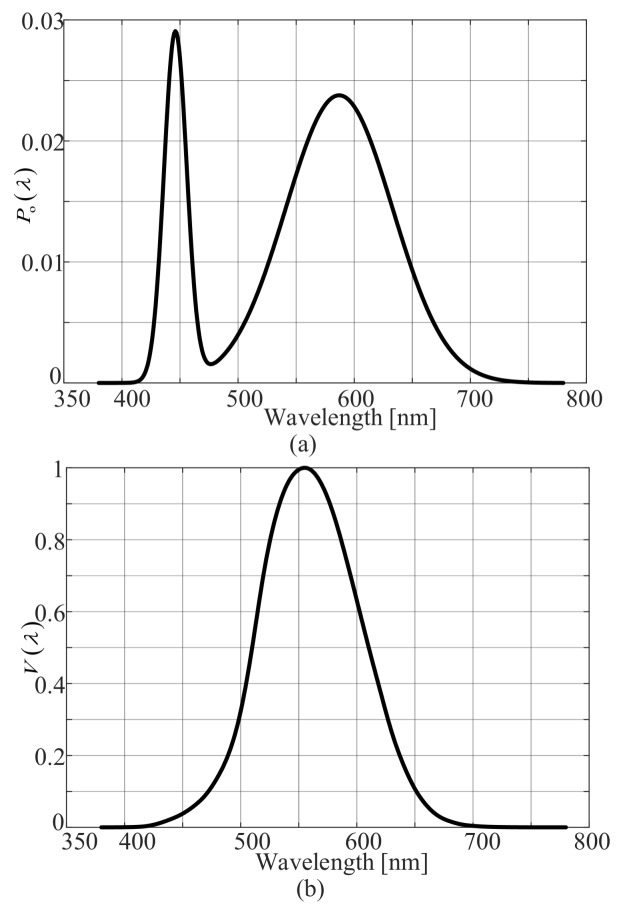
(**a**) The normalized optical spectrum of a typical white LED; (**b**) The normalized Photopic curve.

**Figure A2:**
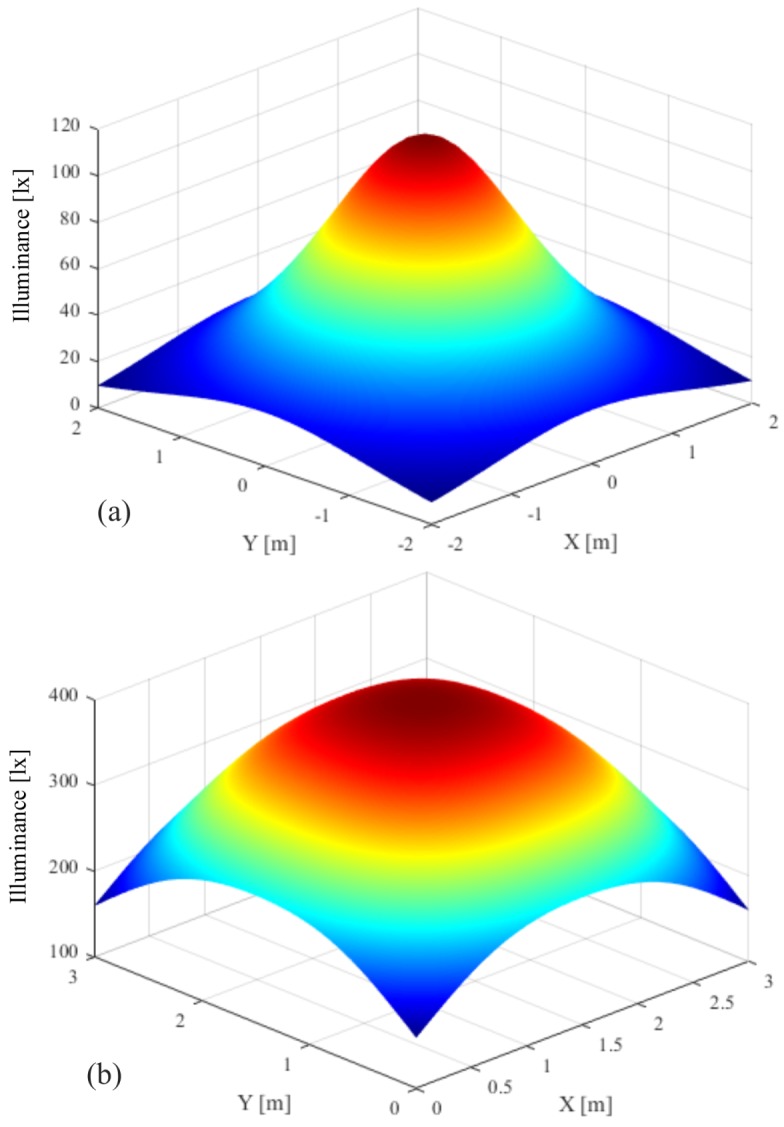
Luminance level in (**a**) an area equipped with a single transmitter with a unity optical power; (**b**) a room with four luminaires when each luminaire is transmitting 1.57 W of optical power.

## Appendix A. Transmit Optical Power

In VLP, LED luminaires are used for both illumination and localization. Therefore, it is important to ensure that the transmitted optical power translates into the recommended level of illuminance when designing a VLP system. The relation between the optical power and the illuminance depends on the optical power spectrum of the light source and the human eye perception to brightness. In this appendix, we show how the transmitted optical power is chosen to satisfy the illumination requirement in a typical workplace room.

The luminous flux of a light source depends on the spectral power distribution of the light source and is given by

(A1) $$\Phi =P_{\mathrm{opt}}\Phi_{0}$$

where $P_{\mathrm{opt}}$ is the transmit optical power and $\Phi_{0}$ is the optical flux that corresponds to a normalized optical power spectrum, $\int_{380}^{780}P_{o}\left(\lambda \right)\;d\lambda =1$, and it is given by

(A2) $$\Phi_{0}=k_{m}\int_{380}^{780}P_{0}\left(\lambda \right)V\left(\lambda \right)\;d\lambda$$

where $k_{m}=683\;\mathrm{Lm}/w$ is the maximum spectral luminous efficacy at λ=555nm, and $V\left(\lambda \right)$ is the luminosity function, which describes the average spectral sensitivity of human visual perception of brightness. Figure A1a shows the normalized optical spectrum of a typical white LED. The luminosity function is represented by the photopic curve shown in Figure A1b.

The brightness of an area is usually expressed by the illuminance, *E*, in lux, which is the incident luminous flux, Φ, per unit area. The illuminance depends on the radiation pattern of the light source, the direction of the incident light and distance between the light source and the illuminated area. For a Lambertian light source of order *m* with the light coming from $\left(\varphi ,\theta \right)$ and an area that is parallel to the plane on which the light source is located, the illuminance is given by

(A3) $$E=\Phi \times \frac{m+1}{2\pi l^{2}}\cos^{m}\phi \cos\varphi$$

By substituting Equation (A1) in Equation (A3), the transmit optical power of the light source is calculated using

(A4) $$P_{\mathrm{opt}}=\frac{2\pi l^{2}E}{\left(m+1\right)\times \Phi_{0}\times \cos^{m}\phi \cos\varphi }$$

Figure A2a shows the illuminance level for a transmitter with a transmit optical power of 1 W. The illuminance is at maximum at the area directly below the transmitter at 120 lx and it decreases until it reaches zero lx for $\left|x\right|>2$ or $\left|y\right|>2$

According to the Australian illumination standard, AS/NZS 1680.1:2006, in a workplace with simple to moderate illumination requirements, the recommended illuminance is between 160lx and 400 lx [43]. By considering a typical room configuration similar to the room shown in Figure 11. From Equation (3), the transmit optical power required by each luminaire to provide a maximum illuminance of 400 lx at the center of the room at a table height of 0.7 m is 1.57 W. Figure A2b illustrates the distribution of the illuminance level over the room. It demonstrates that the considered transmit optical power is sufficient to provide levels of illuminance that are within the recommended levels over the room.

## Appendix B. Background Light Spectral Density

Background light and its level of illuminance define the amount of shot noise generated at the receiver. In this appendix, we show how the shot noise is calculated based on the normal illumination requirement in an office.

The important parameter in the calculation of the shot noise is the spectral irradiance of the background light in watts per $cm^{2}\cdot \mathrm{nm}$. For a certain level of illuminance, *E*, the spectral irradiance is given by

(A5) $$p_{n}\left(\lambda \right)=\frac{P_{o}\left(\lambda \right)\times E}{\Phi_{0}}\;\times 10^{-4}$$

where $10^{-4}$ is the conversion factor from $1/m^{2}\;$ to $1/cm^{2}\;$. For background illuminance of 400 lx caused by LED light sources, the mean shot noise spectral irradiance is equal to $0.29\;\mu W/\left(cm^{2}\cdot \mathrm{nm}\right)$ which is much smaller than the worst-case value of $6\;\mu W/\left(cm^{2}\cdot \mathrm{nm}\right)$.
